# β-Glucan Chitosan Particle Provides Cross-Protection Against Multi-Drug- Resistant Candida auris

**DOI:** 10.21203/rs.3.rs-7942966/v1

**Published:** 2025-11-24

**Authors:** Shakti Singh, Ashley Barbarino, Eman G. Youssef, Kaustav Das Gupta, Sunna Nabeela, Teclegiorgis Gebremariam, Sondus Alkhazraji, Priya Uppuluri, Garry Ostroff, Ashraf S. Ibrahim

**Affiliations:** The Lundquist Institute for Biomedical Innovation at Harbor-University of California Los Angeles (UCLA) Medical Center; The Lundquist Institute for Biomedical Innovation at Harbor-University of California Los Angeles (UCLA) Medical Center; The Lundquist Institute for Biomedical Innovation at Harbor-University of California Los Angeles (UCLA) Medical Center; The Lundquist Institute for Biomedical Innovation at Harbor-University of California Los Angeles (UCLA) Medical Center; The Lundquist Institute for Biomedical Innovation at Harbor-University of California Los Angeles (UCLA) Medical Center; The Lundquist Institute for Biomedical Innovation at Harbor-University of California Los Angeles (UCLA) Medical Center; The Lundquist Institute for Biomedical Innovation at Harbor-University of California Los Angeles (UCLA) Medical Center; The Lundquist Institute for Biomedical Innovation at Harbor-University of California Los Angeles (UCLA) Medical Center; University of Massachusetts Chan Medical School; The Lundquist Institute for Biomedical Innovation at Harbor-University of California Los Angeles (UCLA) Medical Center

**Keywords:** Candida auris, candidiasis, vaccine, β-glucan particle, cross-reactive

## Abstract

*Candida auris* is a multidrug-resistant fungal pathogen that can survive outside the host, easily spread, and colonize the healthcare environment, medical devices, and human skin. *C. auris* causes serious, life-threatening infections with mortality rates of ~ 60% in immunosuppressed patients. Some isolates of *C. auris* are resistant to virtually all clinically available antifungal drugs. Therefore, alternative therapeutic approaches are urgently needed. *C. auris* cell wall contains β-glucan similar to non-pathogenic yeasts such as *Saccharomyces cerevisiae*. Moreover, cellular proteins are also heavily glycosylated with β−1,3 and β−1,6 glycan structures. Recently, a monoclonal antibody raised against β-glucan was shown to recognize *C. albicans* hyphal-regulated cell wall protein (Hyr1p), which has a closely related family of proteins in *C auris*. We tested β-glucan chitosan particles (GCP) as a vaccine candidate and evaluated the humoral and cellular immune responses. Interestingly, GCP induced robust IgG antibody and Th1/Th2/Th17 immune responses that cross-reacted with purified *C. auris* cell wall. Anti-GCP antibodies recognized the cell walls of *C. auris* isolates from four major clades through binding to cell wall glycoproteins. Mice vaccinated with GCP were protected from disseminated *C. auris* infection compared to placebo mice (40% vs. 0% survival, p = < 0.0001). GCP-vaccinated mice had significantly lower fungal burden than placebo in target organs (kidney, heart, and brain) and fewer fungal abscesses. The mechanism of protection appeared to require antibodies and T-cell activation. These data represent an important step forward toward developing an effective vaccine strategy against multidrug-resistant *C. auris*.

## INTRODUCTION

Discovered over a decade ago, *C. auris* has emerged rapidly in over 40 countries worldwide^1–3^. *C. auris* has independently evolved in six distinct clades (I-VI) isolated from different geographic regions: South Asia, East Asia, Africa, South America, Iran, and Bangladesh and Singapore^4–8^. One of the intriguing features of the *C. auris* is its ability to spread and colonize both inanimate objects and human skin within healthcare settings. This feature poses a significant risk to immunosuppressed patients in contaminated healthcare facilities^9,10^, where *C. auris* can infect the patients through attached invasive medical devices and surgical procedures^11–14^. *In vivo* studies show that hematogenously disseminated *C. auris* infection can result in the invasion of vital organs such as the kidneys, heart, and brain^15,16^. Bloodstream infections caused by *C. auris* are exceptionally challenging to treat and have a staggering mortality rate of approximately 60%^4^. Recently, the widespread use of corticosteroid therapy to manage immunopathological effects related to COVID-19 has led to an increase in COVID-19-associated fungal infections, including *C. auris*^17^.

Multidrug resistance is commonly associated with bacterial rather than fungal infections. However, this perception is changing due to the emergence of drug resistance in fungal pathogens like *C. auris*^18,19^. Almost all *C. auris* clinical isolates are reported to be azole-resistant. The prevalence of polyene resistance among *C. auris* is high and estimated to be 90%^4^. In general, resistance to echinocandins among *C. auris* remains to be low with 7% of all clinical isolates reported to be echinocandin resistant^4^. However, a study from India found out that 37% of the tested clinical isolates had reduced susceptibility to caspofungin acetate (minimum inhibitory concentration [MIC] of ≥ 1 μg/ml)^20^. Regardless of the current frequency of resistance among *C. aruis*, there is an ever-increasing number of clinical isolates demonstrating resistance to all available antifungal drug classes (pan-resistance), rendering their infections untreatable^18,19^. Thus, the U.S. Centers for Disease Control and Prevention (CDC)^20^ and the World Health Organization (WHO) have declared *C. auris* an “urgent threat” to public health^21^. Therefore, clearly alternative strategies for managing *C. auris* infections are needed.

Our research on *Candida albicans* has yielded significant success in developing vaccine-based approaches to control fungal infections. We have identified two *C. albicans* cell surface proteins as potential vaccine candidates: Agglutinin-like sequence-3 protein (Als3p), an adhesin/invasin, and Hyphal-regulated protein-1 (Hyr1p), a neutrophil evasion factor^22–25^. Vaccines containing recombinant N-terminal regions of Als3p or Hyr1p were shown to protect mice against systemic *C. albicans* infections^25–28^. Furthermore, NDV-3A, a vaccine based on the N-terminus of Als3p adjuvanted with Alum, was shown to be safe and protected women against vulvovaginal candidiasis in a phase II clinical trial^29,30^. We identified Agglutinin-like sequence (Als) orthologs and Hyr1/Iff family proteins in *C. auris*, and both NDV-3A and mAb targeting a shared epitope in *C. albicans* Hyr1 protein provided protection against lethal murine infections with *C. auris*^16,31^.

Glucan and chitosan are major components of the fungal cell wall, sharing common structural features among various fungi^32,33^. Monoclonal antibodies raised against glucan have been shown to recognize multiple fungi, including *C. albicans* and *C. auris*^34,35^. Interestingly, these antibodies can cross-react with cell walls, including highly glycosylated Als3p and Hyr1p^35^. Fungal β-glucans are well-known pathogen-associated molecular patterns (PAMPs) recognized by pattern recognition receptors (PRRs) on the host cells, stimulating an immune response^36^. Due to this reason, Fungal β-glucans has been exploited as novel vaccine adjuvants^37–39^, that induce a robust Th-17 response critically needed for protection against fungal infections. Currently, GCP is used as an adjuvant for an experimental *Coccidioides posadasii* vaccine candidate^38^.

Interestingly, while investigating potential adjuvants for the protein subunit vaccine against invasive candidiasis (IC), mice vaccinated with GCP alone (adjuvant-only control) were protected against lethal disseminated *C. auris* infection. Further investigation showed that the GCP induced strong cross-reactive antibody and T-cell responses against *C. auris* cell wall, protecting against *C. auris* infection in mice. We further explored the mechanism of protection of GCP vaccination against *C. auris*.

## RESULTS

### β-glucan particles and glucan-chitosan particles induced robust humoral and cellular immunity.

**Figure 1:**
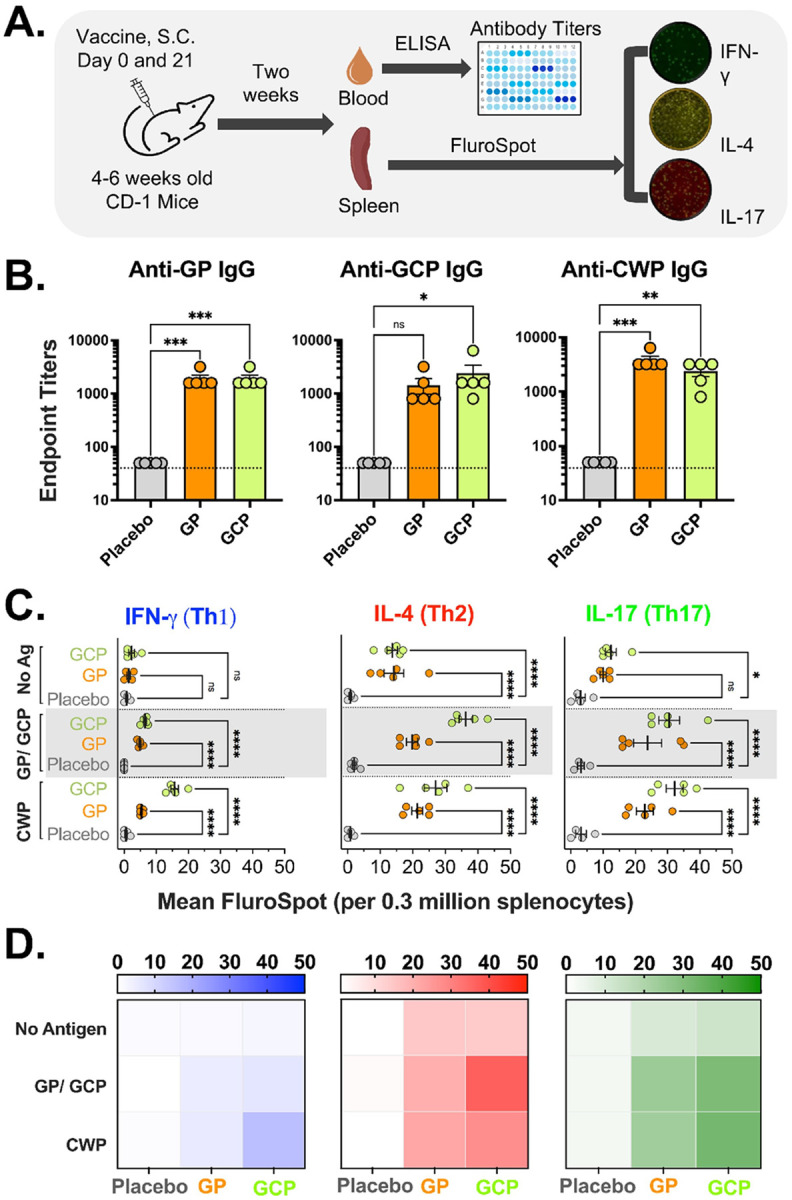
GCP and GP vaccination induces robust humoral and cellular immunity. **(A)** 4–6 weeks old ICR CD-1 mice (n=5/group) were vaccinated with GP, GCP or placebo (PBS, diluent) on day 0 and 21. Two weeks after the booster immunization, blood and spleen were collected to evaluate the immune responses. (**B)** IgG antibody titers were determined using ELISA plates coated with vaccine antigens (GP and GCP) and *C. auris* cell wall protein (CWP). Serial dilutions of serum samples were added to the ELISA plates to determine the end-point titers. Data are presented as Mean + SEM titers. (**C-D)** Spleen from each mouse was homogenized to get splenocyte single-cell suspension. RBCs were removed by RBC lysis, and 3×10^5^ splenocytes were added to each well of the FluroSpot assay plate coated with a mixture of anti-mouse IFN-g (blue), IL-4 (red), and IL-17 (green) capture antibodies. The splenocytes were stimulated with a final 10 ug/ml of the following antigens: GP, GCP, *C. auris* CWP PMA/Ionamycin (assay positive control, not shown). After 24 hours of incubation with antigens, the plates were developed for FluroSpots using detection antibodies, per the manufacturer’s instructions. Assay plates were scanned, and spots were counted using CTL analyzer. Mean+SE or mean antigen-specific spot-forming units (SFU) for each mice group (n=5 mice) are presented in graphs (C) or heat maps (D). *P<0.05, **p<0.005, ***p<0.0005, ****p<0.00005.

Mice were vaccinated with β-glucan particles (GP) or β-glucan chitosan particles (GCP) through the subcutaneous (S.C.) route on days 0 and 21. Two weeks after the final vaccination, GP, GCP, and *C. auris* cell wall proteins (CWP)-specific IgG and T cell responses were determined using ELISA and FluroSpot, respectively. For the T cell responses, we determined antigen-specific Th1, Th2, and Th17 immune responses by quantifying IFN-γ, IL-4, and IL17-producing immune cells, respectively. Splenocytes from placebo, GP, or GCP-vaccinated mice were stimulated with GP (for GP-vaccinated mice) or GCP (for GCP-vaccinated mice) or *C. auris* CWP for 24 hours in a FluroSpot plate coated with specific cytokine capture antibodies (Fig. 1A). Mean IgG titers (± standard error [SE]) and the mean fluoro spot counts (± SE) were plotted and compared.

GP and GCP induced robust anti-GP and anti-GCP IgG antibody responses (p < 0.05). GP-vaccinated mice showed 1920 ± 320 (mean ± SE) anti-GP and anti-GCP IgG titers, whereas GCP-vaccinated mice had 1440 ± 466 anti-GP and 2400 ± 1012 anti-GCP IgG titers. IgG antibodies induced by GP and GCP vaccination were also quantified against *C. auris* cell wall proteins (CWP). Strikingly, both GP and GCP-vaccinated mice induced robust cross-reactive anti-*C. auris* CWP IgG antibodies (3840 ± 640 and 2400 ± 506, respectively) (Fig. 1B).

Furthermore, GP and GCP vaccination induced robust GP and GCP-specific Th1, Th2, and Th17 immune responses (p < 0.05). In contrast, the placebo mice did not induce detectable GP, GCP, or CWP-specific T-cell responses. Specifically, GP and GCP vaccinations induced significant antigen-specific Th1 responses vs. placebo (p < 0.0001). Interestingly, GCP vaccination induced two times more CWP-specific Th1 responses than GP. Also, both GP and GCP-vaccinated mice produced significantly higher IL-4 and Th17 responses, even without *ex vivo* antigen stimulation, potentially due to non-specific T-cell activation in these mice. The induction of Th2 and Th17 responses from splenocytes of GP and GCP vaccinated mice was further enhanced when the cells were ex vivo stimulated with GP, GCP, or CWP. Finally, GCP vaccination showed consistently higher antigen-specific Th1, Th2, and Th17 responses compared to GP vaccination. T cell responses in GP and GCP mice were biased towards Th2 and Th17 types. (Figs. 1C and 1D). Collectively, these data show that both GP and GCP induce robust *C. auris* CWP-specific immunity.

### Anti-GCP IgG antibodies recognize C. auris isolates from all major clades, reduce biofilm formation, and enhance macrophage OPK activity.

**Figure 2:**
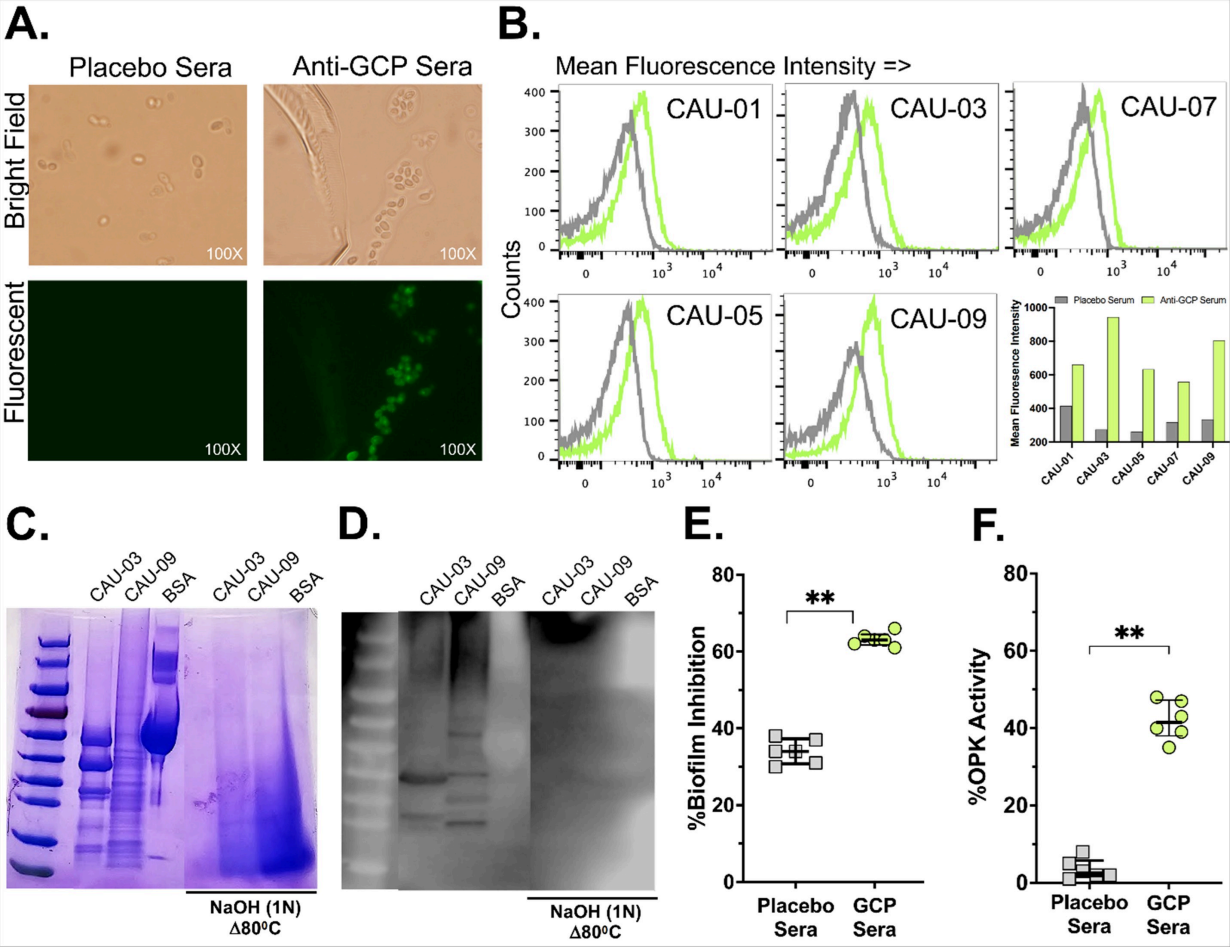
Binding of anti-GCP IgG antibodies to C. auris isolates from different clades and their functional activity. Binding of anti-GCP IgG antibodies to *C. auris* isolates from different clades and their functional activity. The binding capacity of anti-GCP IgG antibodies to *C. auris* isolates (CAU-01, CAU-03, CAU-05, CAU-07, and CAU-09), representing different clades, was assessed by incubating yeast cells with serum from GCP- or placebo-vaccinated mice. Bound IgG was detected using Alexa Fluor 480-labeled anti-mouse IgG antibodies. This was followed by: (**A**) Fluorescence imaging of the stained yeast cells, in which *C. auris* cells exhibited green fluorescence, indicated successful binding of anti-GCP IgG to the cell surface. In contrast, cells incubated with placebo sera showed no such fluorescence. (**B**) Quantitative analysis by flow cytometry, in which binding of anti-GCP IgG to clinical *C. auris* isolates from the four major clades was demonstrated by a rightward shift in fluorescence intensity peaks compared to the placebo group, along with significantly higher mean fluorescence intensity (MFI) values. (**C-D**) Cell wall proteins (CWP) extracted from *C. auris* (CAU03 (Clade III) and CAU09 (Clade I)), Bovine serum albumin (BSA) or β-glucan chitosan particles (GCP) were either left untreated or were hydrolyzed using NaOH (1N, 80°C, 10 mins). Resultant proteins/peptides were separated by SDS-PAGE and either stained with Coomassie Brilliant Blue (**C**) or immunoblotted using sera from mice injected with GCP and infected with *Candida auris* (**D**). **C** and **D** are representative immunoblot from two independent experiments. (**E**) Anti-biofilm activity of anti-GCP and placebo sera was assayed in 96-well plates using mouse serum from GCP- or placebo-vaccinated mice. Biofilm was quantified by XTT assay after 24 hours of incubation. (**F**) Effect of anti-GCP-antibodies on mouse macrophages OPK of *C. auris* (CAU-09) (1:2.5 target: effector) for 2 hours. Statistical significance was determined by the Mann-Whitney Test. The p-values <0.05 were considered significant (**p<0.005).

Considering vaccination with GCP induced superior *C. auris*-CWP-specific immune responses, we further explored the *C. auris* cross-reactive potential of anti-GCP IgG. We studied the binding capacity of anti-GCP IgG antibodies to the *C. auris* isolates (CAU-01, CAU-03, CAU-05, CAU-07, CAU-09) representing different clades by incubating the cells with serum from GCP or placebo-vaccinated mice. Bound IgG antibodies were detected by mouse anti-IgG antibodies labeled with Alexa-Fluor 480. This was followed by imaging of the stained yeast cells and quantification of the extent of binding by flow cytometry. The anti-GCP IgG antibodies recognized the cell surface of *C. auris*, while sera obtained from placebo mice did not bind to the yeast cells (Fig. 2A). Further, flow cytometry analysis of the stained yeast cells showed that anti-GCP IgG antibodies bound clinical isolates of *C. auris* from four major clades as represented by a shift in the peaks towards the right side vs. placebo and significantly higher mean fluorescent intensities of yeast with anti-GCP IgG vs. placebo (Fig. 2B).

To determine whether anti-GCP IgG antibodies recognize only glucan structures or also require protein components for binding to the *C. auris* cell wall, we isolated cell wall fractions from strains CAU-03 and CAU-09. A portion of each preparation was treated with 1 N NaOH at 80°C to remove alkaline-soluble polysaccharides from proteins^40^. These samples were then analyzed by SDS-PAGE followed by Western blotting using anti-GCP or placebo sera. The anti-GCP sera detected multiple bands in both CAU-03 and CAU-09 cell wall preparations, but not in bovine serum albumin (BSA), which served as a non-specific protein control. Notably, this reactivity was completely lost in the NaOH-treated samples, indicating that the anti-GCP antibodies specifically recognize carbohydrate moiety of protein structures (glycans) of the *C. auris* cell wall. Additionally, the distinct banding patterns observed between CAU-03 and CAU-09 suggest heterogeneity in the target glycoproteins across these two different clades (Fig. 2C–D).

Many cell surface proteins of *Candida* are involved in cell adhesion and biofilm formation^41–44^. Hence, we tested whether the anti-GCP IgG antibody that bound to *C. auris* also influenced biofilm formation, a trait that is essential for the yeast survival during infection. We incubated *C. auris* CAU-09 in the presence of anti-GCP or placebo containing murine sera and allowed biofilm formation for 24 h prior to comparing the % biofilm formation of *C. auris* without any added serum. While sera obtained from placebo mice resulted in ~ 35% inhibition of biofilm formation, sera obtained from mice vaccinated with GCP resulted in ~ 60% inhibition of biofilm formation (i.e. >25% increase in biofilm inhibition) (p < 0.005) (Fig. 2E).

We also determined the opsonophagocytic capability of the anti-GCP sera. *C. auris* was incubated with sera obtained from mice vaccinated with GCP or placebo for opsonization, followed by incubation with the murine-derived primary macrophages. The anti-GCP sera resulted in > 20-fold increase in macrophage killing when compared to sera obtained from placebo (2% OPK activity for sera obtained from placebo mice vs. 40% OPK activity for sera obtained from GCP vaccinated mice, p < 0.005) (Fig. 2F).

### Vaccinating with GCP protects mice against lethal hematogenously disseminated C. auris infection.

**Figure 3:**
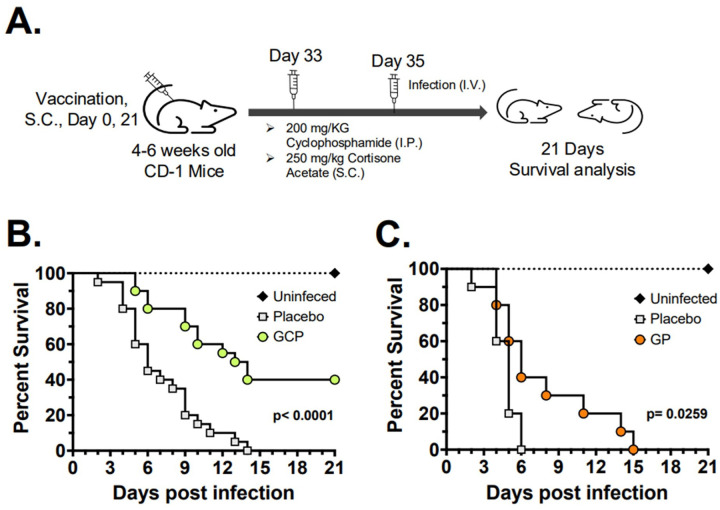
GCP vaccination protects mice against hematogenously disseminated *C. auris* infection. 4–6 weeks old male ICR CD-1 mice were vaccinated with 200 μg/mouse of GCP or GP placebo (PBS, diluent) on days 0 and 21. The mice were immunosuppressed with 200 mg/kg cyclophosphamide (I.P.) and 250 mg/kg cortisone acetate (S.C.) on day 33. The mice were infected with a lethal dose (2 × 10^7^ cells/mouse) of *C. auris* through tail vein injections on day 35 and observed for 21 days for their survival (**A**). Survival analysis of GCP vaccinated mice (n=20/group) (**B**). Survival analysis of GP vaccinated mice (n=10/group) (**C**). Survival data were plotted and analyzed by the Mantel-Cox survival analysis. The p-values <0.05 were considered significant.

GP and GCP vaccination induced robust antibody and T-cell responses targeting *C. auris* cell wall, inhibited *C. auris* biofilm formation, and increased OPK activity of macrophages *in vitro*. Thus, we investigated whether vaccination with either GCP or GP can protect mice against lethal hematogenously disseminated *C. auris* infection. Four-six-week-old ICR CD-1 mice (n ≥ 9) were subcutaneously vaccinated with two doses of GP, GCP, or diluent (Placebo) administered on days 0 and 21. On day 33, mice were immunosuppressed with cyclophosphamide + cortisone acetate, then infected on day 35 with intravenous administration of 5×10^7^ cells of *C. auris* (CAU-09)/mouse. Mice were monitored for moribundity up to 21 days with moribund mice humanely euthanized (Fig. 3A).

Mice vaccinated with GCP were significantly protected against lethal *C. auris* infection, with 40% survival and ~ 14 days of median survival time (MST) vs. 0% survival and 6 days of MST of placebo mice (Fig. 3B). The GP vaccination modestly prolonged time to complete moribundity with 100% mortality of vaccinated mice reaching on day 15 vs. 100% mortality of placebo mice on Day 6 (p = 0.0259) (Fig. 3C).

Next, we investigated whether GCP vaccine-mediated protection is specific to *C. auris* or is effective against lethal hematogenously disseminated *C. albicans* infection. We infected immunocompetent mice, that have been vaccinated as above, with a lethal inoculum of *C. albicans* (2 × 10^5^ cells/mouse) intravenously after two weeks of the final and second dose of vaccination. In this model, GCP vaccination did not provide any survival benefits against *C. albicans* infection, suggesting that the GCP vaccine-mediated protection is *C. auris-*specific (**Figure S1**).

Finally, because GCP-containing adjuvants have innate immune activation properties, we investigated whether the therapeutic administration of GCP benefits acute *C. auris* infection. We infected immunosuppressed mice with a lethal *C. auris* inoculum and then vaccinated the mice with one dose (day 1 post-infection) or two doses (day 1 and 8 post-infection) of GCP administered subcutaneously. Although the single or dual therapeutic administration of GCP vaccine did not provide significant protection against *C. auris*, 30% and 10% of the mice that have been subjected to dual and single vaccination, respectively, survived the infection by day 21, versus 0% for placebo (unvaccinated mice) (**Figure S2**).

### Vaccination with GCP reduces C. auris tissue fungal burden and attenuates fungal-mediated damage in target organs.

**Figure 4:**
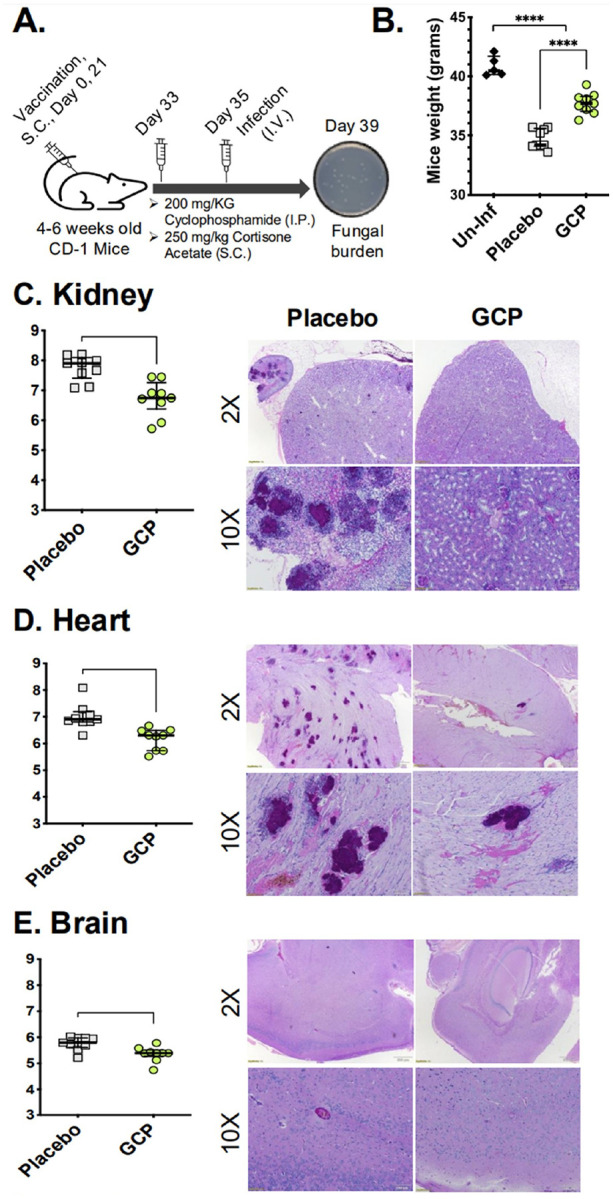
GCP vaccination reduces *C. auris* burden and improved overall health. (**A**) 4–6 weeks old ICR CD-1 mice (n=9–10/group) were vaccinated with 200 μg/mouse of GP, GCP, or placebo (PBS, diluent) on days 0 and 21 and infected on day 35 (two days after the final vaccination). The mice were immunosuppressed two days before infection with a lethal dose (5 × 10^7^ cells/mouse) of *C. auris* through tail vein injections. After day 4 post-infection, mice were weighed (**B**), and euthanized to determine fungal burden in kidney (**C)**, hear(**D**) t, and brain (**E)**. Fungal burden was represented as CFU/gram of tissue and analyzed using the Mann-Whitney test to determine the difference in fungal burden. P values <0.05 were considered statistically significant (**p<0.005, ***p<0.0005, ****p<0.00005). Organs from each group were also analyzed for histopathology by sectioning and staining with Periodic Acid-Schiff (PAS) and imaged by Olympus bright-field microscopy at 2X and 10X (**C-E**, right panels).

We investigated whether vaccination with GCP reduces *C. auris* burden in the kidney (primary target organ), and the heart and brain (secondary target organs) (Fig. 4A). Infected mice experienced a significant loss in body weight after four days of infection with *C. auris* compared to placebo-treated mice (p < 0.0005), highlighting the severity of the infection. Interestingly, mice that received the GCP vaccine demonstrated a markedly lower degree of weight loss than their placebo counterparts, indicating that GCP vaccination effectively reduced the severity of *C. auris* infection (Fig. 4B; p < 0.0005). Furthermore, GCP-vaccinated mice showed a significant reduction in fungal burden, with approximately a 1.0-log, 0.5-log, and 0.3-log decrease in fungal load observed in the kidney, heart, and brain tissues, respectively (p < 0.005) (Fig. 4C–E). This reduction in fungal burden was further supported by histopathological analyses, which revealed that vaccinated mice had fewer and smaller fungal lesions across these target organs. Moreover, the tissue architecture in GCP-vaccinated mice was better preserved, with less evidence of tissue damage compared to the placebo group. Histological sections showed more organized and intact structures in vaccinated animals, while placebo-treated mice displayed disrupted and damaged tissues (Fig. 4C–E). Collectively, these findings indicate that vaccinating mice with GCP not only limits fungal proliferation but also protects against tissue damage, thereby significantly mitigating the impact of *C. auris* infection.

### Humoral immunity and T helper cells are essential for GCP vaccine-mediated protection against lethal hematogenous C. auris infection

**Figure 5:**
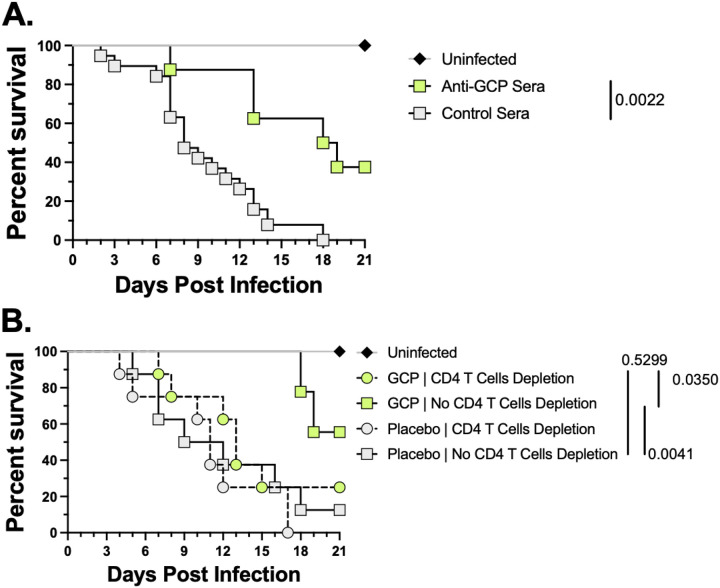
Immune mechanism of vaccine-mediated protection. (**A**.) Naïve 4–6 weeks ICR CD-1 mice were infected with a lethal dose (target inoculum 5 × 10^7^ cells/mouse) of *C. auris* through intravenous injection, while given two doses at 1 hour and 7 days post-infection of i.p. injection of anti-GCP IgG antibodies isolated from previously GCP vaccinated mice or placebo control mice sera. (**B.**) 4–6 weeks old ICR CD-1 mice were vaccinated with GCP or placebo (PBS, diluent) on days 0 and 21, followed by injection of anti-CD4 or isotype-matched antibodies (0.2 mg / mouse) intraperitoneally on day 32 and 35, and 200 mg/kg cyclophosphamide (I.P.) and 250 mg/kg cortisone acetate (S.C.) on day 33. All mice were infected with 5 × 10^7^ *C. auris* yeast cells/mouse intravenously on day 35. Survival data were plotted and analyzed by the Mantel-Cox survival analysis. P values <0.05 were considered statistically significant.

To determine the role of antibodies in GCP-vaccine-mediated protection against lethal *C. auris* infection, we conducted passive immunization experiments. We collected serum from GCP- or PBS-vaccinated mice, then pooled and passively transferred the anti-GCP or placebo sera intraperitoneally into immunosuppressed ICR CD-1 mice infected with a lethal dose of *C. auris*. Anti-GCP sera was given 1 hour and a repeat dose 7 days post-infection. Survival of mice was followed for 21 days. Mice that received anti-GCP sera showed a significant 40% survival rate with 18.5 days of MST vs. mice that received placebo sera, which had 0% survival with 8 days MST (P = 0.0022, Fig. 5A).

To evaluate the role of cellular immunity, we vaccinated mice with GCP or placebo on days 0 and 21, depleted their CD4^+^ T cells on day 32. CD4 + T cell depleted mice were then immunosuppressed with cyclophosphamide and cortisone acetate on day 33 prior to infecting then with *C. auris* on day 35. We compared the survival of these CD4^+^ T cell depleted mice to the survival of normal mice with GCP- or placebo-vaccinated mice without. As expected, GCP vaccination provided significant protection against mortality as earlier (55% survival with > 21 days of MST) compared to placebo group. In contrast, mice lacking CD4^+^ T cells lost the protective benefit of GCP vaccination and showed significantly lower survival than GCP-vaccinated mice with intact CD4^+^ T cells (P = 0.0350; Fig. 5B). Furthermore, we observed no significant difference in survival between the CD4^+^-depleted GCP-vaccinated mice and the placebo group (P = 0.5299; Fig. 5B). Collectively, these data highlight the critical role of antibodies and CD4^+^ T helper cells in GCP-vaccine-mediated protection against lethal hematogenously disseminated *C. auris* infection.

## DISCUSSION

*C. auris* exhibits a broad spectrum of drug resistance mechanisms (including mutations in ERG genes involved in ergosterol synthesis and upregulation of efflux pumps) present across all clades, making it challenging to develop new drug variants within existing antifungal classes^5,45–48^. Thus, novel antifungal interventions with distinct mechanisms of action or alternative immune-based prophylactic or therapeutic strategies appear to be the most effective approach.

β-glucan has been shown to induce protective effects against fungal infections, such as *Pneumocystis pneumonia*^49^ and *Mycobacterium bovis*^50^, through mechanisms involving macrophage activation and antibody cross-reactivity with fungal cell wall carbohydrates. Due to their ability to activate macrophages and drive humoral and Th2/Th17 immune responses, β-glucan is being developed as a vaccine adjuvant, especially for fungal infections^37–39^. In our *C. albicans* Als3p and Hyr1p dual antigen-based vaccine approach against *Candida* infections, we explored the potential use of GCP and GP particles as adjuvants and unexpectedly discovered a protective effect of GCP particles (without *Candida* antigens) against *C. auris*. This unexpected result prompted us to further explore GCP-induced cross-protection against *C. auris* lethal infection.

Our study demonstrates that β-glucan particles (GP) and glucan-chitosan particles (GCP) serve as effective vaccine platforms that induce robust humoral and cellular immunity against *C. auris*, a multidrug-resistant fungal pathogen of global concern. Notably, GCP vaccination consistently outperformed GP across multiple immunological and protective endpoints, highlighting the combined effect of chitosan and β-glucan.

We show that GP and GCP vaccinations elicit strong antigen-specific IgG responses, including cross-reactive antibodies against *C. auris* cell wall proteins (CWPs). The significantly higher IgG titers observed in GCP-vaccinated mice compared to GP alone underscore the enhanced immunogenicity imparted by the chitosan component. Moreover, these antibodies were capable of recognizing *C. auris* isolates from all four major clades, indicating broad-spectrum reactivity, a crucial feature for a globally relevant vaccine.

In addition to antibody production, both GP and GCP vaccinations elicited strong antigen-specific Th1, Th2, and Th17 responses, which was skewed toward Th2 and Th17 polarization. GCP consistently induced higher cytokine responses, even without *ex vivo* antigen stimulation. This suggests that GCP formulations not only stimulate adaptive immunity but also potentiate a heightened state of innate immune activation. Importantly, CWP-specific Th1 responses, critical for fungal clearance^51–53^, were significantly stronger in GCP-vaccinated mice. This Th1 dominance correlated with enhanced protection *in vivo*, further underscoring the functional relevance of this response profile.

Our findings demonstrate that anti-GCP IgG antibodies robustly recognize *C. auris* cells across multiple clinical isolates representing four major clades, as evidenced by both confocal microscopy and flow cytometry. Further SDS-PAGE analysis and NaOH hydrolyzation studies revealed that anti-GCP antibodies highly likely recognize glycosylated CWP. Of importance, the anti-GCP antibodies recognized heterogenic glycans among *C. auris* strains, potentially due to different patterns and densities of the glycosylation. Further investigations are required to identify these cell wall proteins and their expression profile across different *C. auris* clades, which may have implications for novel diagnostic and therapeutic strategies targeting this emerging pathogen.

The functionality of anti-GCP antibodies was evidenced by their ability to inhibit *C. auris* biofilm formation and enhance opsonophagocytic killing by macrophages, key mechanisms implicated in fungal clearance and attenuation of virulence. This functional immune response translated into robust *in vivo* protection, Notably, this protection was specific to *C. auris*, as it did not extend to *C. albicans*, reinforcing the antigen-specific nature of the immune response.

Further mechanistic insights were gained through passive immunization and CD4^+^ T cell depletion studies. Transfer of anti-GCP sera conferred significant protection, highlighting the pivotal role of humoral immunity. Similarly, CD4^+^ T cells were indispensable for GCP-mediated protection, as their depletion completely abrogated the survival benefit. This observation is consistent with prior studies emphasizing the protective role of CD4^+^ T helper cells, particularly the Th17 subset, in immunity against *C. auris*^54^. Notably, while Th17 responses are associated with fungal clearance, a Th1-skewed CD4^+^ T cell response has been shown to exacerbate *C. auris* skin infection, likely due to IFN-γ–mediated suppression of IL-17–driven protective mechanisms^55^. This pathogenic Th1 bias contrasts with the immune profile induced by GCP vaccination, which appears to favor Th2/Th17 polarization over Th1, potentially contributing to its protective efficacy. Together, these findings confirm that both arms of adaptive immunity antibody-mediated and CD4^+^ T cell-dependent are essential for vaccine efficacy. Interestingly, while GCP showed modest survival trends when administered therapeutically postinfection, the lack of statistical significance suggests that prophylactic rather than therapeutic use is likely the most effective approach for this formulation. However, future studies should also investigate the potential use of GCP + antifungal in a therapeutic administration.

In conclusion, our results suggest that GCP could serve as a standalone vaccine or a potent adjuvant in a broader vaccine strategy against *C. auris*. GCP can induce durable and functional humoral and cellular immunity with broad clade coverage that translates into significant protection against lethal *C. auris* infection.

## MATERIALS AND METHODS

### *Candida* culture and strains

*C. auris* strains CAU-01 (Clade II), CAU-03 (Clade III), CAU-05 (Clade IV), CAU-07 (Clade I), and CAU-09 (Clade I) were obtained from the Centers for Disease Control and Prevention (CDC). *C. albicans* (SC5314) and *C. auris* strains were cultured overnight in Yeast Extract Peptone Dextrose (YPD) broth in a shaker incubator at 30°C and 200 rpm. The following day, the yeast cells were centrifuged at 4000 RPM for 10 minutes at 4°C, followed by a triple wash with 1X phosphate-buffered saline (PBS). Subsequently, the yeast cells were resuspended in 1X PBS and counted using a hemocytometer.

In serum antibody binding experiments, 5 × 10^6^ cells/ml *C. auris* cells were cultured under physiological conditions in RPMI-1640 medium, which was enriched with L-glutamine and 10% fetal bovine serum at 37°C on a shaker for 75 minutes.

### Vaccine and immunization

GP and GCP were developed as adjuvants derived from the cell walls of nonpathogenic yeasts, specifically *Saccharomyces cerevisiae* for GP and *Rhodotorula mucilaginosa* for GCP^37,39^. GPs and GCPs are hollow structures of fungal cell walls, predominantly consisting of β−1,3-D-glucans. GP and GCP were prepared as per the methods described earlier^37^. The outbred male ICR CD-1 aged 4–6 weeks mice (n=5 mice/group) were immunized with 200 μg/0.1 ml/mouse of GP or GCP on days 0 and 21. Vaccine diluent (1X Phosphate buffer saline, pH 7.2) was used as placebo. Two weeks after the final vaccination (day 35), mice were euthanized to collect sera and spleens for analysis or infected for the vaccine efficacy determination^31^.

### IgG antibody titer determination

Sera were used to assess the IgG endpoint titers against GP, GCP, or *C. auris* cell wall proteins (CWP) using ELISA. The plates were coated with 5 μg/ml of GP, GCP, or CWP extracted from *C. auris* CAU-09^56^ in 1X PBS and incubated overnight at 4°C. The next day, the plates were washed with 1X wash buffer containing 1X PBS and 0.05% Tween-20 three times. The serum samples were serially diluted in 1X wash buffer containing 1% bovine serum albumin (BSA) and added to the wells in duplicates. After 1 hour of incubation at room temperature, the plates were washed, and anti-mouse IgG labeled with HRP was added to each well at a 1:1000 dilution in 1X wash buffer containing 1% BSA. After 1 hour of incubation, the plates were washed as above, and TMB blue substrate solution was added to each well. After 10–30 minutes of incubation, the HRP-substrate reaction was stopped by 1N sulfuric acid, and the absorbance was measured at 450 nm. The endpoint titer was determined by the reciprocal of the highest dilution having OD450 greater than blank + 2*standard deviation^57^.

### FluroSpot Assay

Mouse spleens were individually processed to obtain splenocyte cell suspension, as described earlier^31^. The splenocytes were counted, and cell density was adjusted to 0.3 × 10^6^ cells/0.1 ml with serum-free culture media (CTL Serum-free media). One day before the experiment, the assay plates were coated with IFN-g, IL-4, or IL-17 capture antibodies and incubated at 4°C overnight. The next day, the plates were washed, and GP, GCP, and CWP antigen suspension was added at a final 10 μg/ml prepared in serum-free CTL media. No antigens and mitogens cocktail containing PMA/Ionomycin (Cell Stimulation Cocktail, eBioscience^™^) was used as a negative and positive stimulation controls. Splenocytes were added at 0.3 × 10^6^ cells/well and the plates were incubated at 37°C for 24 hours in stationary condition. After the incubation, the plates were washed with 1X PBS containing 0.05% Tween-20 and developed by CTL Triple Color FluroSpot assay reagents, including detection antibodies with green, red or yellow dyes, as per the kit manual (Catlog# mT3004F, mT02, mT38, mT31, ImmunoSpot, Cleavland, OH). The frequencies of antigen-specific T cells were determined by subtracting the counts from unstimulated wells (no antigen) from the counts in the GP, GCP or CWP-stimulated wells^58^.

### Flow cytometry

*C. auris* yeast cells were cultured as described above. Yeast cells at 2 × 10^6^ cells/tube were incubated with the 1: 200 diluted sera in 1X PBS with 1% BSA solution and incubated for 1 hour at room temperature. After the incubation, the cells were washed three times with 1x PBS containing 0.05% Tween-20, and then 0.1 ml of Alexa Fluor 488 labeled anti-mouse IgG detection antibodies were added at 1:100 dilution. After 1 hour of incubation at room temperature, the cells were washed three times and resuspended in 300 μl of PBS. All procedures were performed at 4°C to prevent *C. auris* replication. The stained cell suspension was imaged under confocal microscopy or transferred to tubes for flow cytometry analysis. Twenty thousand events/samples were acquired using BD FACSymphony (BD Sciences, Franklin Lakes, NJ, USA), and data was analyzed using FlowJo software (Version 10)^16,31^.

### Extraction of Cell Wall Proteins (CWP) from *Candida auris*

An overnight culture of *Candida auris* was washed three times with cold PBS. After the final wash, the cell pellet was resuspended in Tris-HCl (ph 7.5) containing protease inhibitor cocktail. Fungal cells were lysed using Lysing Matrix Y (MPBio, Cat: SKU:1169600-CF) in a bead beater (15 times for 40 sec with 1min intervals in between, with cells on ice). Cell wall fraction (pellet) was separated by centrifuging the lysate at 3000 g for 10 min. The cell wall fraction was washed four times with ice cold water followed by an additional four times wash with ice-cold saline solution (1M NaCl, 1 mM PMSF). The cell wall fraction was resuspended in protein extraction buffer (50 mM Tris-HCl, pH 8, 2% SDS, 100 mM EDTA, 10 mM DTT and 40 mM b-mercaptoethanol). Resuspended cell wall fraction was incubated at 100°C for 5 min. The fraction was again centrifuged twice at 3000 g for 10 min, while the supernatant containing intracellular fraction was discarded each time. The cell wall fraction was washed three times with distilled water and resuspended in PBS containing 0.1% Tween-20 and protease inhibitor cocktail. The cell wall fraction was again subjected to bead beating (10 times for 20 sec with 30 sec intervals in between, with cells on ice). Resultant cell wall proteins were quantified using BCA assay (ThermoFisher Scientific, Cat: 23235).

### SDS-PAGE and Immunoblotting

Approximately 20 mg protein (*Candida auris* CWP, BSA and GCP) were treated with 1N NaOH and heated at 80°C for 10 min. The pH was neutralized the resultant hydrolyzed proteins were mixed with Laemmli buffer (ThermoFisher Scientific, Cat: J61337.AD) containing reducing agent. Native proteins and hydrolysed proteins were then separated using SDS-PAGE and the polyacrylamide gel was stained with Coomassie Brilliant Blue. For immunoblotting, the proteins were transferred on to a nitrocellulose membrane and probed with sera from mice injected with GCP and infected with *Candida auris*. HRP-conjugated mouse IgG (ThermoFisher Scientific, Cat: 31430) was used as the secondary antibody for signal detection using chemiluminescent ECL substrate (ThermoFisher Scientific, Cat: 34577).

### Biofilm formation assay

Biofilms were developed in 96-well polystyrene microtiter plates, as previously described^16,57^. Briefly, *C. auris* cells were added at 2 × 10^5^ cells/50 μl/well (n=6/test group) to 50 μl of 1:200 diluted anti-GCP mouse serum or isotype matched control IgG1 containing wells and incubated at 37°C for 24 hours to allow the adhesion and biofilm formation. The next day, the wells were gently washed twice with 1X PBS, and the extent of biofilm formation was quantified by XTT assay (450 nm). Data are presented as % biofilm reduction ([1-OD450 of wells with anti-GCP sera/OD450 of wells with placebo sera] *100)^16,57,59^.

### OPK assay.

To assess the OPK activity of anti-GCP antibodies, *C. auris* cells were incubated with 1:200 diluted mouse anti-GCP or placebo sera in a round-bottom 96-well assay plate at 4°C for 1 hour to allow opsonization. Murine macrophages were isolated from the intraperitoneal cavity of naïve mice and adjusted to a concentration of 2.5 × 10^6^ cells/mL. After the initial incubation, the assay plate was transferred to 37°C for 10 minutes, followed by the addition of macrophages at a 1:2.5 yeast to phagocytes ratio to each well. The plate was then incubated for 2 hours at 37°C in a CO_2_ incubator to facilitate phagocytosis. Following incubation, 0.1 ml of 1:100 diluted cell mixtures from the above plates were plated on YPD agar for viable *C. auris* enumeration after overnight incubation at 37 °C. *C. auris* cells without macrophages and *C. auris* with macrophages but without antibody served as non-OPK controls. The percent killing of *C. auris* was calculated using the following formula: {1- [CFUs from wells with (sera + *C. auris* + macrophages)/average CFU in tubes with (*C. auris* + macrophage)]}*100^16,57,59^.

### Mice infection and treatment

For *in vivo* efficacy evaluation, the vaccinated male ICR CD-1 mice (on days 0 and 21) were immunosuppressed with 200 mg/kg cyclophosphamide intraperitoneal and 250 mg/kg cortisone acetate subcutaneous injections on days −2 relative to infection (day 33). To prevent bacterial superinfection, enrofloxacin (at 50 μg/ml) was added to the drinking water. These mice were infected intravenously with 5 × 10^7^ cells of *C. auris* (CAU-09)/mouse/0.2 mL. For *C. albicans* infection, the immunocompetent male ICR CD-1 mice (n=10/group) were infected with 2 × 10^5^ yeast cells intravenously. Both infections were performed two weeks after the final vaccination (day 35)^31^.

For fungal burden determination, mice were infected as above, weighed, and euthanized on day 4 post-infection. The kidneys, hearts, and brains of mice were used for fungal enumeration. The homogenized tissues were 10-fold diluted and quantitatively cultured on YPD plates. Plates were incubated at 37°C for 48 hours before enumerating CFU/gram of tissue. The representative mouse organs were fixed in 10% zinc-buffered formalin, embedded in paraffin, sectioned, and stained with the Periodic Acid Schiff (PAS) stain. Stained tissue sections were imaged on an Olympus microscope^16,31^.

For the GCP therapeutic treatment study, the 4–6 weeks old naïve immunosuppressed male ICR CD-1 mice (n=10/group) were infected with *C. auris* as above and treated with 0.1 mg GCP after 1 and 8 days of infection. For these studies survival followed for 21 days post-infection, served as an endpoint.

### Adoptive sera transfer studies

Naive 4–6 weeks old naive immunosuppressed ICR CD-1 mice (n=10/group) were infected with *C. auris* as previously described. After 1 hour and 7 days of infection, mice were intraperitoneally injected with 0.1 ml of pooled anti-GCP or placebo sera collected from previously vaccinated mice. Mice were monitored for their survival 21 days post-infection^31^.

### CD4 depletion studies

ICR CD-1 mice (n=10/group) were vaccinated with GCP- or placebo on day 0 and 21, followed by CD4 T cell depletion by administering 0.2 mg/mouse anti-CD4 IgG2b (clone GK1.5, Bio X Cell) or isotype-matched control (LTF-2, Bio X Cell) antibodies on day 32 and 35, and administration of 200 mg/kg cyclophosphamide and 250 mg/kg cortisone acetate on day 33. The mice were infected on day 35 with lethal intravenous *C. auris* (CAU-09) inoculum as described earlier. The CD4 T cell depletion was verified on day 39 by staining splenocytes and lymph node cells with anti-CD3 APC (BD Pharmigen, Cat #BDB565643) and anti-CD4 Alexa Fluor 700 antibodies (Biolegend, Cat #100536) and analyzing the frequency of CD4+ T cell using flow cytometry (BD FACS Symphony). Infected mice were monitored for their survival 21 days post-infection^31^.

### Statistical analysis

Differences in survival studies were analyzed by the Log-Rank test for overall survival and with Mantel-Cox comparisons for median survival times. All other comparisons were conducted with the Mann-Whitney test. P values <0.05 were considered statistically significant.

## Supplementary Material

Supplementary Files

This is a list of supplementary files associated with this preprint. Click to download.

- SFig2.tiff
- SFig1.tiff
- GCPCAUSupplimentaryFile1.211.04.2025Final.docx

## Data Availability

The data are available in the main text or the supplementary materials of this manuscript.
